# BMMSC-sEV-derived miR-328a-3p promotes ECM remodeling of damaged urethral sphincters via the Sirt7/TGFβ signaling pathway

**DOI:** 10.1186/s13287-020-01808-2

**Published:** 2020-07-16

**Authors:** Hanke Zhang, Jiayu Huang, Jiaying Liu, Yanhui Li, Ying Gao

**Affiliations:** Department of Obstetrics and Gynecology, Union Hospital, Tongji Medical College, Huazhong University of Science and Technology, Wuhan, 430022 China

**Keywords:** Stress urinary incontinence, Bone marrow mesenchymal stem cells, Small extracellular vesicles, miRNA, TGF-β1, Extracellular matrix

## Abstract

**Background:**

Stress urinary incontinence (SUI) is a common and bothersome condition. Invasive surgery will always be considered after conservative treatment fails, but the rates of postoperative complications and long-term recurrence are high. Thus, a new treatment strategy is still needed. In recent years, bone marrow mesenchymal stem cells (BMMSC) have shown great promise for SUI treatment. The therapeutic effects of BMMSC on SUI are achieved mainly by paracrine pathway signaling molecules, such as small extracellular vesicles (sEV). sEV are recognized as essential mediators of cell-to-cell communication. However, the therapeutic effects and detailed mechanisms of BMMSC-derived sEV in SUI remain mostly unexplored.

**Methods:**

The effects of BMMSC-sEV on extracellular matrix (ECM) metabolism were assessed in vitro and in vivo. In a SUI rat model, TGF-β1 signaling was examined with or without BMMSC-sEV stimulation. sEV miRNAs were deeply sequenced, and the most likely miRNAs were evaluated as mediators of the TGF-β1 signaling pathway.

**Results:**

BMMSC-sEV enhanced the synthesis of ECM components, including elastin, collagen I, and collagen III, and improved urethral function. Furthermore, BMMSC-sEV activated TGF-β1 signaling in primary fibroblast cells and in rat urethras. Several differentially expressed miRNAs were identified in the BMMSC-sEV. Bioinformatics analysis and in vitro studies showed that BMMSC-sEV miR-328a-3p can be transferred from BMMSC to fibroblasts and can regulate the Sirt7/TGF-β1 signaling pathway.

**Conclusion:**

BMMSC-sEV promote ECM remodeling of damaged urethral sphincters by transferring miR-328a-3p to regulate the Sirt7/TGF-β1 signaling pathway.

## Background

Stress urinary incontinence (SUI) is characterized by the involuntary loss of urine upon increases in abdominal pressure, such as that caused by exercise or coughing [1]. SUI is a type of pelvic floor dysfunction that is widespread in females and has an overall incidence of 14.9% [2]. At present, surgery is the most frequently used treatment for patients with SUI after ineffective conservative therapy, but the long-term recurrence rate after surgery is quite high. The recurrence rate can be more than 50%, especially when its pathogenesis involves a functional defect of the urethral sphincter. To avoid postoperative recurrence and reoperation, mesh has been used to strengthen the repair. However, mesh implantation has caused many complications, such as recurrence, pain, and infection [3]. Therefore, a new effective treatment for SUI is still needed.

One of the primary pathogeneses of SUI is the change in extracellular matrix (ECM) composition in the urethral sphincter, and this change is mainly manifested by the reduction in elastin fibers and collagen fibers and the breaking and disordered alignment of elastin fibers [4–8]. As the primary cytokine regulating ECM metabolism, transforming growth factor β1 (TGF-β1) promotes the expression of elastin, collagen, and fibronectin by phosphorylating Smad2 and Smad3 [9, 10]. Therefore, the TGF-β1 signaling pathway has been an essential target for treating SUI [11, 12].

It has been widely reported that stem cells can motivate the ECM reconstruction of the urethral sphincter to promote the recovery of urethral function in SUI [13–16]. Among the stem cells that have been studied, bone marrow mesenchymal stem cells (BMMSC) are the most widely used stem cells in tissue repair and regenerative medicine due to their universal source, self-renewal capacity in vitro, and low immunogenicity. The rationale for BMMSC therapy is increasingly recognized as a secretion (paracrine) rather than a differentiation mechanism in many diseases, such as cardiac injury, aberrant immune response, and SUI [17–19]. Recently, several groups have demonstrated that small extracellular vesicles (sEV) are secreted agents mediating BMMSC paracrine therapeutic efficacy. sEV are small membrane vesicles released from various cell types that transfer molecules from donor cells to recipient cells, including proteins, mRNAs, and miRNAs [20, 21]. Among the signaling molecules in sEV, miRNAs have been the most studied because of their functions in regulating targeted gene expression. Increasingly, studies have found that sEV miRNAs repress the translation of multiple targeted mRNAs to regulate different signaling pathways in recipient cells [20, 22, 23].

In the present study, we sought to examine whether BMMSC-sEV promote ECM remodeling and damaged urethra repair and to further probe the underlying molecular mechanism of sEV-delivered miRNAs in the therapeutic process.

## Methods and materials

### Cell isolation and culture

BMMSC were isolated from the femurs and tibia bone marrow of Sprague-Dawley (SD) rats aged between 3 and 4 weeks. All animals were maintained at the experimental animal center of Huazhong University of Science and Technology. Animal experiments were approved by the Institutional Animal Care and Use Committee (IACUC) of Huazhong University of Science and Technology and were performed in accordance with the guidelines and standards of IACUC. The rat femur and tibia bones were isolated in sterile conditions and washed three times with PBS containing 1% penicillin/streptomycin. The bone marrow cavity was exposed by cutting off both ends of the femur and tibia. The bone cavity was rinsed 10 times with Dulbecco’s modified Eagle’s medium: Nutrient Mixture F-12 (DMEM/F12, HyClone, USA) containing 10% fetal bovine serum (FBS, Gibco, USA) and 1% penicillin/streptomycin.

Fibroblasts were acquired from the periurethral vaginal wall tissues of 5 female SUI patients. These patients were aged between 30 and 60 years old, met the diagnostic criteria for SUI, and were scheduled for surgical treatment without other types of incontinence, neurogenic bladder, pelvic floor organ prolapse, and acute urinary tract or vaginal infections. The Ethics Committees of Union Hospital, Tongji Medical College, Huazhong University of Science and Technology approved this study (No. 2019-S013), and patients’ written informed consent was obtained before surgery. The specimens were placed in sterile PBS and immediately transferred to the laboratory for processing. Primary vaginal wall fibroblasts were isolated and cultured as described in previous studies [24, 25]. First, the periurethral vaginal wall tissues were washed with PBS containing 1% penicillin/streptomycin, and the connective tissues were cut away. Then, the tissues were placed epidermis side down and covered with a Trypsin-EDTA solution (Biosharp, China) in 6-well plates at 4 °C. After 12 h, the epidermis was peeled off, and the dermis was cut into 1-mm^3^ pieces and digested by 0.1% type 1 collagenase (Gibco, USA) at 37 °C for 2 h. The mixture obtained by preceding procedures was filtered through a 40-μm filter (Falcon, USA) and centrifuged at 1000 rpm for 5 min to obtain the cell pellet. Afterward, the cell pellet was cultured in DMEM/high glucose (HyClone, USA) containing 10% FBS (Gibco, USA) and 1% penicillin/streptomycin.

The BMMSC and fibroblasts were cultured at 37 °C in 5% CO_2_. After 48 h, the culture medium was replaced with fresh medium to remove unattached cells. After that, the medium was replaced every 3 days. For the following experiments, BMMSC and fibroblasts were used at passages 3–5.

Normal rat kidney 52E cell (NRK-52E) is isolated from rat renal epithelium, and it is a common immortalized cell line as a control in cell studies due to the definite cellular characteristics. In addition, our preliminary experiment showed that NRK-52E-sEV could not improve ECM deposition in fibroblasts in vitro. Thus, NRK-52E cells were chosen as the control for the subsequent research. NRK-52E and human embryonic kidney 293T cells (HEK-293T) were obtained from the Central Laboratory (Wuhan Union Hospital, China).

### Cell identification

At passage 3, 1 × 10^5^ human primary fibroblasts were seeded in 12-well plates precoated with a cover glass for 48 h. Then, the cells on the glass were fixed with paraformaldehyde and stained with hematoxylin and eosin (HE) or vimentin. The markers of BMMSC were confirmed by flow cytometry at passage 3. BMMSC were collected and incubated with anti-CD90.1-APC (eBioscience, USA), anti-CD34-FITC (NOVUSBIO, USA), and anti-CD45-FITC (Invitrogen, USA) at 4 °C for 30 min. Then, the labeled cells were washed twice with PBS by centrifugation and resuspended in PBS containing 0.5% BSA. The fluorescence was observed by flow cytometry (Beckman Coulter, USA), and the cell morphology was identified by a microscope.

### sEV isolation and identification

The sEV were isolated as described by Li et al. [26]. The sEV-free FBS was isolated as described by Théry et al. [27]. Before sEV isolation, FBS was centrifuged at 120,000*g* for 12 h, then the supernatant was obtained as sEV-free FBS. BMMSC and NRK-52E were cultured in DMEM/F12 containing 10% sEV-free FBS for 48 h. Then, the culture medium was centrifuged at 300*g* for 10 min to eliminate dead cells and was centrifuged at 3000*g* for 20 min to remove cell debris. The obtained supernatant was concentrated by an Ultra-15 centrifugal filter unit (Millipore, USA) and then centrifuged at 13,000*g* for 30 min to remove the microvesicles. Afterward, the supernatant was centrifuged at 120,000*g* for 70 min to concentrate the sEV. The pellet was washed with PBS by repeating the centrifugation conditions of the last step and was resuspended in a small volume of PBS. For the following experiments, the sEV were stored at − 80 °C. The morphology of sEV was identified by transmission electron microscopy (Hitachi H7500 TEM, Japan). The diameter was measured by NanoSight (Malvern Panalytical, UK). The surface markers of sEV, CD9 and CD81, were detected by a western blot. The protein content was quantified using a bicinchoninic acid protein assay kit (Beyotime, China).

### Fibroblast uptake of PKH26-labeled sEV

sEV were labeled with 1 μM PKH26 (Sigma, USA) at room temperature for 5 min and then washed with PBS by centrifuging at 120,000*g* for 70 min to remove unbound PKH26. After that, the labeled pellet was resuspended in PBS and added to human fibroblasts cultured in a 35-mm confocal dish (10 μg per dish). After 24 h, cells were washed with PBS and fixed in 4% paraformaldehyde. Next, the cell nuclei were stained with 4′,6-diamidino-2-phenylindole (DAPI, Servicebio, China), and the cytoskeleton was stained with phalloidin (Sigma, USA). Images were acquired using a laser scanning confocal microscope.

### sEV treatment of fibroblasts

Fibroblasts (2 × 10^5^) were seeded in 6-well plates. After 24 h, the culture medium was replaced with DMEM/F12 containing 10% sEV-free FBS. Immediately following, the cells were treated with PBS, different concentrations of BMMSC-sEV (1 μg/ml, 5 μg/ml, 10 μg/ml, or 20 μg/ml), or NRK-52E-sEV. After 48 h, the cells were collected for protein extraction.

### Western blot analysis

The protein samples (30 μg per lane) were separated by 10% SDS-PAGE, transferred to a polyvinylidene fluoride membrane (PVDF, Millipore, USA), and then blocked with 5% nonfat milk at room temperature for 1 h. The membrane was incubated overnight at 4 °C with primary antibodies, including those against GAPDH (1:2000, Proteintech, China), CD9 (1:1000, Abcam, USA), CD81 (1:1000, Abcam, USA), elastin (1:1000, Abcam, USA), collagen I (1:1000, Abcam, USA), collagen III (1:1000, Abcam, USA), Smad2/3 (1:1000, Cell Signaling Technology, USA), p-Smad2/3 (1:1000, Cell Signaling Technology, USA), TGF-β1 (1:1000, Abcam, USA), and Sirt7 (1:1000, Cell Signaling Technology, USA). Next, the membrane was incubated with an HRP-conjugated antibody (1:4000, Servicebio, China) at room temperature for 1 h and detected using an ECL chemiluminescence detection kit (Servicebio, China) with a BioSpectrum 600 Imaging System (UVP, CA, USA). The band density was determined by the ImageJ software (National Institutes of Health, Bethesda, MD, USA).

### SUI rat model and treatment

A rat model of SUI was established via transabdominal urethrolysis as described in our previous study [28] and in a study by Rodriguez et al. [29]. Twenty-five female SD rats aged 8 weeks were randomized into 5 groups (*n* = 5/group): group 1, control group; group 2, sham group; group 3, NRK-52E-sEV group; group 4, BMMSC group; and group 5, BMMSC-sEV group. The experiments that were performed with each group are shown in Table 1. Groups 2–5 were anesthetized with an intraperitoneal injection of 10% chloral hydrate. An abdominal incision was made, and the urethra was detached circumferentially from the anterior vaginal wall and pubic bone. Then, bilateral ovariectomies were performed to eliminate the influence of the estrus cycle on ECM metabolism and to simulate an estrogen-deficient state. Finally, the abdominal skin was closed with wound clips.

**Table 1:** The experiments performed on rats

	Control (*n* = 5)	Sham (*n* = 5)	NRK-52E-sEV (*n* = 5)	BMMSC (*n* = 5)	BMMSC-sEV (*n* = 5)
Urethrolysis	−	+	+	+	+
Periurethral injection	−	100 μl PBS	100 μg NRK-52E-sEV	2 × 10^6^ BMMSC cells	100 μg BMMSC-sEV
Frequency of injection	−	Once per week	Once per week	Once	Once per week

After 3 weeks, group 4 was injected once in the urethral wall with 2 × 10^6^ BMMSC cells that had been resuspended in 100 μl DMEM/F12. Group 2 was injected in the urethral wall with 100 μl PBS once a week. Group 3 and group 5 were given 100 μg NRK-52E-sEV and 100 μg BMMSC-sEV once a week in the urethral wall, respectively. The sham group, NRK-52E-sEV group, and BMMSC-sEV group were injected for a total of 5 weeks.

### Leak point pressure measurement

We used the vertical-tilting table method for measuring the leak point pressure (LPP) to evaluate urethral muscle function in each group via the method described by Conway et al. [30]. After 5 weeks of injection, the rats were anesthetized by 10% chloral hydrate. Through a small midline incision in the lower abdomen, a PE catheter was inserted into the bladder and secured. First, the rats were placed in the prone position, and their spinal cords were transected at T8–T9 sections. Then, the rats were taped to a tilting plate and fixed in the vertical position. After that, a 50-ml syringe (without a plunger and filled with methylene blue saline) was connected to the bladder catheter by a three-way cock and secured to a measuring rod. Starting from 0 cm, the height of the injector was artificially increased 2–3 cm every 2 min to fill the bladder, until urinary leakage occurred at the urethral meatus. The LPP was the bladder pressure (measured by the transducer) at which urinary leakage was observed. The LPP test was repeated at least three times for each animal.

### Tissue collection and staining

After the LPPs were measured, the rats were immediately euthanized, and the urethras and bladders were then collected. The proximal part of the urethra was embedded in paraffin, and the transverse tissue was sliced into sections. Subsequently, tissue sections were stained with HE, elastic van Gieson (EVG), and Sirius red. EVG staining was used to detect the expression level of elastic fibers, and Sirius red was used to evaluate the expression levels of the collagen fibers. The remaining tissue was used for protein extraction. The thickness of the smooth muscle layer in HE staining was measured by the NDP.view 2 software (Hamamatsu Photonics K.K., Japan).

### Immunofluorescence staining

The tissue sections were incubated with an anti-α-SMA antibody (1:100, Abcam, USA) overnight at 4 °C. Subsequently, the sections were incubated with the secondary antibody at room temperature for 1 h. DAPI was used to stain the nuclei. Images were acquired using a laser scanning confocal microscope. The area fraction of red fluorescent signals was determined by the ImageJ software.

### In vivo tracking experiment

Nine female SD rats aged 8 weeks were randomly divided into 3 groups (*n* = 3/group): group 1 was periurethrally injected with 100 μl PBS, group 2 was injected with 100 μl DiI, and group 3 was injected with 100 μl DiI-labeled sEV (1 μg/μl). Fluorescence images were taken on days 1, 2, 3, 5, 7, 10, and 14 using the in vivo imaging system (Bruker, German). The images were analyzed by the Bruker MI SE 7.2 software.

### sEV miRNA sequencing and analysis

Total RNA was extracted from purified BMMSC-sEV and NRK-52E-sEV. Small RNA library construction and sequencing were conducted by OE Biotech Co., Ltd. (Shanghai, China). Differentially expressed miRNAs were identified with the threshold of *p* value < 0.05. The *p* value was calculated with the DEG algorithm in the R package for experiments with biological replicates and with the Audic-Claverie statistic for experiments without biological replicates. The targets of differentially expressed miRNAs were predicted by using miRanda, with the parameter as follows: S ≥ 150 ΔG ≤ − 30 kcal/mol and a strict 5′ seed pairing. Gene Ontology (GO) functional enrichment analysis and Kyoto Encyclopedia of Genes and Genomes (KEGG) pathway enrichment analysis of differentially expressed miRNA target genes were performed using R based on the hypergeometric distribution. The differentially expressed miRNAs were further verified by qRT-PCR.

### Dual-luciferase reporter assay

According to bioinformatics prediction, the Sirt7-WT, Sirt7-MUT, miR-328a-3p mimic, miR-148a-5p mimic, and miR-125a-5p mimic sequences were synthesized by Qijing Biological Technology Co., Ltd. (Hubei, China). Sirt7-WT or Sirt7-MuT was inserted into a pmirGLO luciferase vector. HEK-293T (1 × 10^5^) cells were seeded into 12-well plates for 24 h. Then, Sirt7-WT, Sirt7-MuT, or pmirGLO was cotransfected with the miR-328a-3p mimic, miR-148a-5p mimic, miR-125a-5p mimic, or negative control (NC) mimic into HEK-293T cells by using Lipofectamine 3000 reagent (Invitrogen, USA). After 48 h, the luciferase activities were detected by a Dual-luciferase Reporter System (Promega, USA).

### Cell transfection

The miR-328a-3p inhibitor was synthesized by Qijing Biological Technology Co., Ltd. (Hubei, China). Human fibroblasts (2 × 10^5^) were seeded in 6-well plates for 24 h. When the cell density reached 50%, 50 nM miR-328a-3p mimic or 100 nM miR-328a-3p inhibitor was transfected into the cells by using Lipofectamine 3000 reagent (Invitrogen, USA). BMMSC-sEV (10 μg/ml) were added to cells after miR-328a-3p inhibitor transfection. After 48 h, cells were harvested for western blot analysis.

### Statistical analysis

All data are expressed as the means ± SEMs from three different experiments. All data were analyzed by the Prism 8 software (GraphPad Software, CA). Statistical significance was analyzed using Student’s *t* test or one-way and two-way ANOVA, when appropriate, with Tukey’s post hoc test for multiple comparisons. A difference was considered statistically significant when *p* < 0.05.

## Results

### Cell and sEV identification

At passage 3, most of the human fibroblasts were long, spindle-shaped, and arranged in clusters as observed under an inverted microscope (Additional file 1: Fig. S1A). In HE-stained sections, the cells were large, flat, and elongated. The cell nuclei were round or oval (Additional file 1: Fig. S1B); vimentin staining showed that almost 100% of fibroblasts had a brown-yellow cytoplasm, while the nuclei were not colored (Additional file 1: Fig. S1C).

At passage 3, BMMSC had adherent growth ability, and most of the morphology was spindle-shaped and fusiform (Additional file 1: Fig. S1D). Flow cytometry detection showed that BMMSC were strongly positive for CD90 (99.5%) but persistently negative for CD34 (0.16%) and CD45 (0.06%) (Additional file 1: Figure S1Ea-c). These properties were consistent with the criteria described by Dominici et al. [31], which indicated that BMMSC were successfully isolated.

**Fig. 1:**
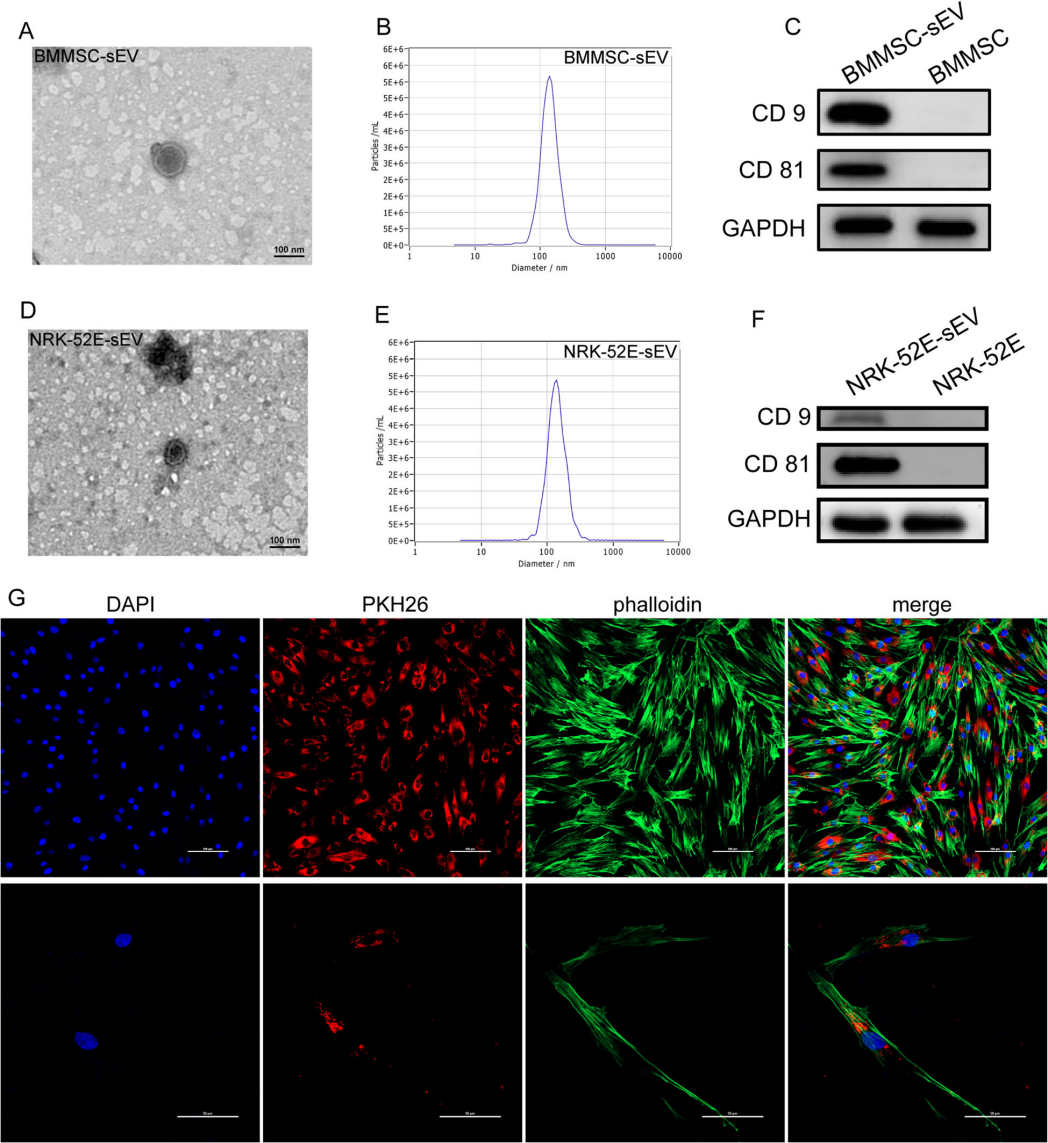
Characterization and internalization of sEV. **a**, **d** TEM showed that the sEV presented with the typical morphology. Scale bar, 100 nm. **b** NTA revealed that the peak diameter of BMMSC-sEV was 138.8 nm. **c**, **f** The western blot results showed that CD9 and CD81 were enriched in sEV but were undetectable in the cells. **e** The peak diameter of NRK-52E-sEV was 134.7 nm. **g** The laser scanning confocal images showed that fibroblasts internalized PKH26-labeled sEV after 24 h. The nuclei were stained blue, the sEV were stained red, and the cytoskeleton was stained green. Scale bars, 100 μm and 50 μm

Transmission electron microscopy showed that the purified sEV had a typical cup- or sphere-shaped morphology (Fig. 1a, d). Nanoparticle tracking analysis (NTA) showed that the peak diameter of BMMSC-sEV was 138.8 nm (Fig. 1b) and the peak diameter of NRK-52E-sEV was 134.7 nm (Fig. 1e). Western blotting demonstrated that all of the particles expressed sEV special surface marker proteins, including CD9 and CD81 (Fig. 1c, f). All these results confirmed that the fibroblasts, BMMSC, and sEV were successfully isolated.

### Fibroblasts internalize PKH26-labeled sEV

The laser scanning confocal images showed that red fluorescent signals were substantially detected in the endochylema of the fibroblasts that were incubated with sEV for 24 h (Fig. 1g), verifying that sEV could be internalized by fibroblasts.

### BMMSC-sEV promote ECM remodeling of fibroblasts

**Fig. 2:**
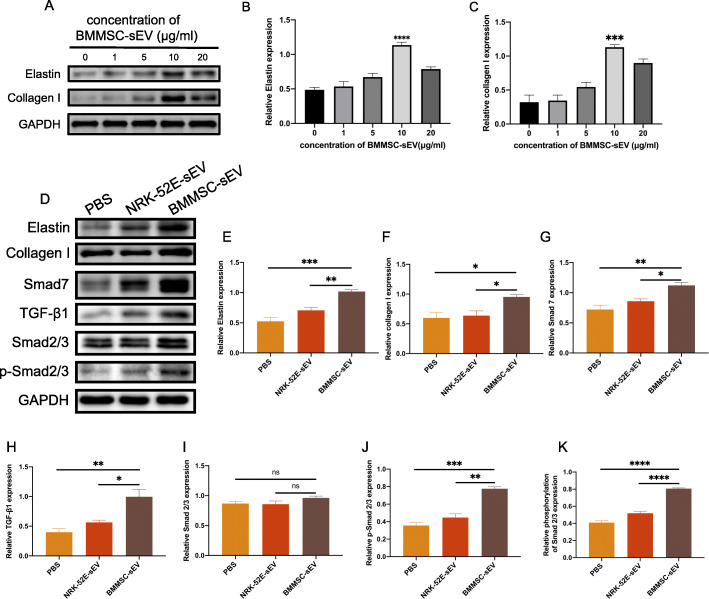
Therapeutic effects of BMMSC-sEV in fibroblasts. **a**–**c** The expression levels of elastin and collagen I in primary human fibroblasts treated with different concentrations of BMMSC-derived sEV. **d**–**j** The expression levels of elastin, collagen I, Smad7, TGF-β1, Smad2/3, and p-Smad2/3 in fibroblasts that were treated with PBS, NRK-52E-sEV (10 μg/ml), or BMMSC-sEV (10 μg/ml) for 48 h. **k** The phosphorylation level of Smad2/3. *p < 0.05, **p < 0.01, ***p < 0.001, ****p < 0.0001

To determine the optimal concentration of BMMSC-sEV that can achieve the best therapeutic effect, we treated fibroblasts with PBS or different concentrations of BMMSC-sEV (1 μg/ml, 5 μg/ml, 10 μg/ml, and 20 μg/ml). The results showed that fibroblasts treated with 10 μg/ml BMMSC-sEV had the highest expression levels of elastin and collagen I (Fig. 2a–c). Therefore, 10 μg/ml sEV was chosen for the following experiments in vitro.

Next, the therapeutic effects of BMMSC-sEV and NRK-52E-sEV were compared in vitro. After incubation for 48 h, western blot analyses showed that compared with PBS and NRK-52E-sEV treatment, BMMSC-sEV treatment significantly enhanced the protein levels of elastin and collagen I in human primary vaginal fibroblasts. However, no significant differences were detected between the PBS group and the NRK-52E-sEV group (Fig. 2d–f). These results confirm that BMMSC-sEV promote ECM remodeling of fibroblasts.

It is recognized that the TGF-β1/Smad signaling pathway plays a critical regulatory role in the metabolism of ECM [32, 33], so we detected the expression level of TGF-β1 and the phosphorylation level of Smad2/3. The results showed that the expression level of TGF-β1 and the phosphorylation level of Smad2/3 were significantly upregulated in the BMMSC-sEV group (Fig. 2d, h–k), which indicated that BMMSC-sEV promote ECM remodeling via the TGF-β1 signaling pathway. It is noteworthy that the expression of Smad7, an inhibitor of TGF-β1 signaling, increased with BMMSC-sEV treatment (Fig. 2d, g).

### In vivo tracking

Fluorescence images showed that the fluorescence signal intensity in DiI-labeled sEV-injected rats and DiI-injected rats mainly converged in the periurethral tissue (Additional file 2: Fig. S2A). During the first week, the fluorescence signal intensity in DiI-labeled sEV-injected rats decreased slowly. On day 7, only 39.44% of the fluorescence was eliminated. However, 96.14% of the fluorescence signal intensity in DiI-labeled sEV-injected rats disappeared after 10 days. Then, no fluorescence signal was detected in DiI-labeled sEV-injected rats on day 14. The fluorescence signal intensity in the DiI group was barely attenuated from day 1 to day 14. There was no fluorescence signal in the PBS group (Additional file 2: Fig. S2A, B). Based on the above evidence, we decided to inject sEV once a week to maintain the effective concentration.

### BMMSC-sEV enhance the function of the damaged urethral sphincter in vivo

**Fig. 3:**
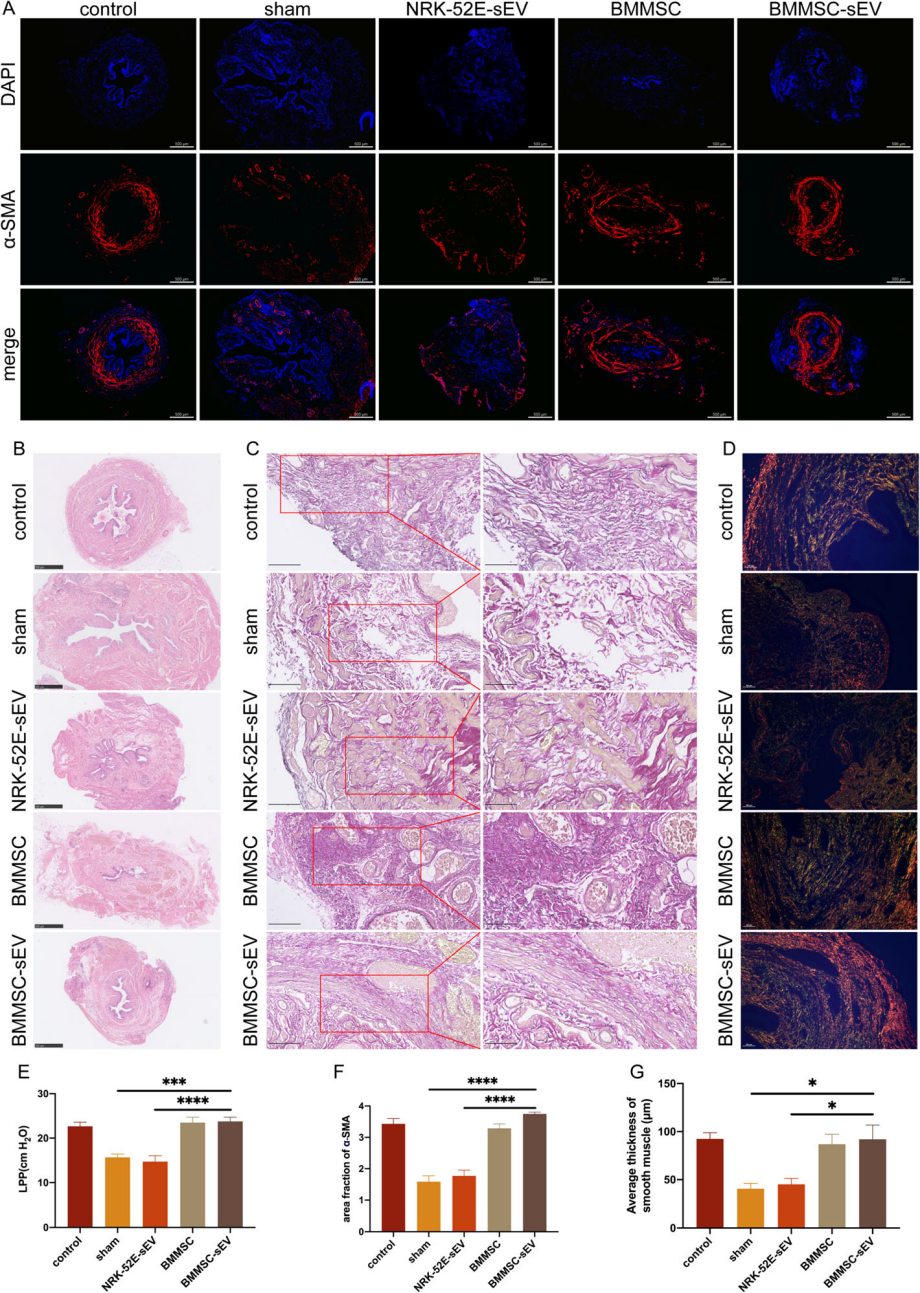
BMMSC-sEV restore urethral function and structure in SUI rats. **a** Detection of the urethral muscle layer by α-SMA staining. The smooth muscle was stained red. Scale bar, 500 μm. **b** HE staining of the urethral sections. Scale bar, 500 μm. **c** The elastic fibers were visualized by EVG staining. Elastic fibers were stained black. Scale bar, 100 μm, 50 μm. **d** Evaluation of collagen fibers under a polarization microscope by Sirius red staining. Collagen I was stained orange or red. Collagen III was stained green. Scale bar, 100 μm. **e** The mean values of the LPP of the BMMSC-sEV group and the BMMSC group were higher than those of the sham group and NRK-52E-sEV group, respectively, while no differences were detected among the BMMSC-sEV group, the BMMSC group, and the intact control group. **f** The area fraction of red fluorescent signals in α-SMA staining. **g** The average thickness of smooth muscle layers in HE staining. *p < 0.05, **p < 0.01, ***p < 0.001, ****p < 0.0001

After 5 weeks of injection, the LPP of the sham group (mean = 15.69 ± 0.75 cmH_2_O) was lower than that of the intact control group (mean = 22.67 ± 0.92 cmH_2_O), which meant that the SUI rat model was successfully developed. The difference in the LPP between the NRK-52E-sEV group (mean = 14.74 ± 1.33 cmH_2_O) and the sham group was not statistically significant, indicating that NRK-52E-sEV had no therapeutic effect on the damaged urethral sphincter. However, the LPPs of the BMMSC-sEV group (mean = 23.76 ± 0.96 cmH_2_O) and BMMSC group (mean = 23.50 ± 1.22 cmH_2_O) were significantly higher than that of the sham group. There was no significant difference among the BMMSC-sEV, BMMSC, and control groups (Table 2, Fig. 3e). Thus, sEV derived from BMMSC have a similar therapeutic effect as BMMSC on the injured rat urethra.

**Table 2:** LPP of SUI rats

	Control (*n* = 5)	Sham (*n* = 5)	NRK-52E-sEV (*n* = 5)	BMMSC (*n* = 5)	BMMSC-sEV (*n* = 5)
LPP (cmH_2_O)	22.67 ± 0.92	15.69 ± 0.75	14.74 ± 1.33	23.50 ± 1.22	23.76 ± 0.96

### BMMSC-sEV restore the structure of the damaged urethral sphincter in vivo

In the HE-stained sections, the urethral sphincter of the control group was continuous and complete. Compared with those of the control group, the urethral tissues of the sham and NRK-52E-sEV groups showed thinner smooth muscle layers and more broken and incoherent muscle texture. Moreover, the layer of the smooth muscle in rats treated with BMMSC or BMMSC-sEV was significantly thicker, and the broken muscle fibers were improved to a level similar to that in the control group (Fig. 3b, g).

In α-SMA staining of the muscle layer, the area fraction of red fluorescent signals in the BMMSC group and the BMMSC-sEV group was much higher than those in the sham group and NRK-52E-sEV group and was similar to that in the control group (Fig. 3a, f), indicating that the muscular layers in the BMMSC group and the BMMSC-sEV group were significantly thicker than those in the sham group and NRK-52E-sEV group.

EVG staining and Sirius red staining showed that rats in the sham group and NRK-52E-sEV group had fewer, thinner, and more disorganized elastic fibers and collagen fibers than the rats in the intact control group. After treatment, the number, length, and thickness of the elastic and collagen fibers were obviously restored in the BMMSC and BMMSC-sEV groups (Fig. 3c, d).

Taken together, these results show that BMMSC-sEV promote the reconstruction of the SUI rat urethra.

### BMMSC-sEV promote ECM remodeling of the damaged urethral sphincter in vivo

**Fig. 4:**
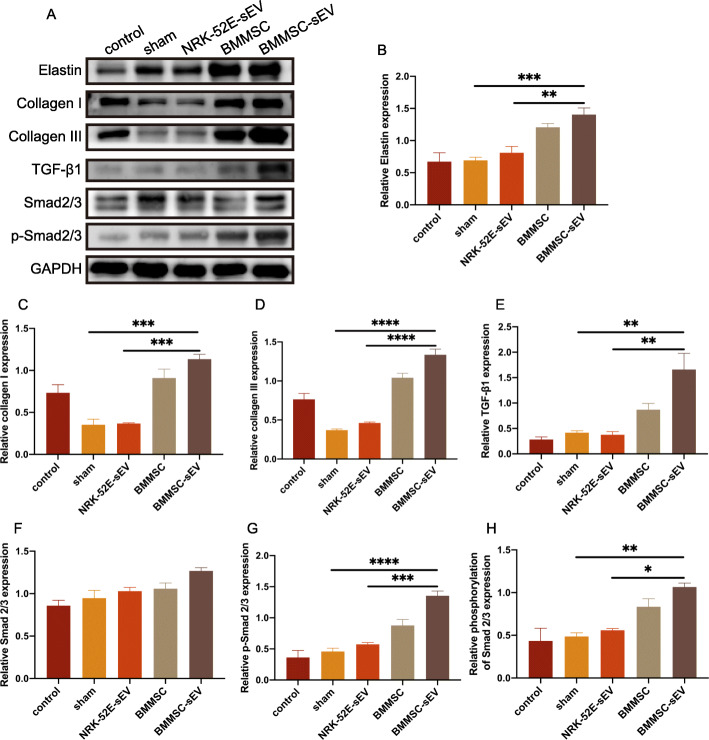
BMMSC-sEV promote urethral ECM remodeling in SUI rats via the TGF-β1 signaling pathway. **a** Western blot analysis showed the expression levels of elastin, collagen I, collagen III, TGF-β1, Smad2/3, and p-Smad2/3 in normal rats and SUI rats treated with PBS, NRK-52E-sEV, BMMSC, and BMMSC-sEV. **b**–**g** Normalized data shown in **a**. **h** Quantified data of the phosphorylation level of Smad2/3. *p < 0.05, **p < 0.01, ***p < 0.001, ****p < 0.0001

The effect of ECM remodeling was examined by western blot analyses. The western blot results showed that compared to PBS treatment and NRK-52E-sEV treatment, BMMSC-sEV treatment significantly increased the protein expression levels of elastin, collagen I, collagen III, and TGF-β1. Notably, both BMMSC-sEV treatment and BMMSC treatment similarly induced the phosphorylation of Smad2/3 (Fig. 4a–h). These results suggested that BMMSC-sEV promoted ECM remodeling of the damaged urethral sphincter via the TGF-β1 pathway.

### Identifying miRNAs enriched in BMMSC-sEV

**Fig. 5:**
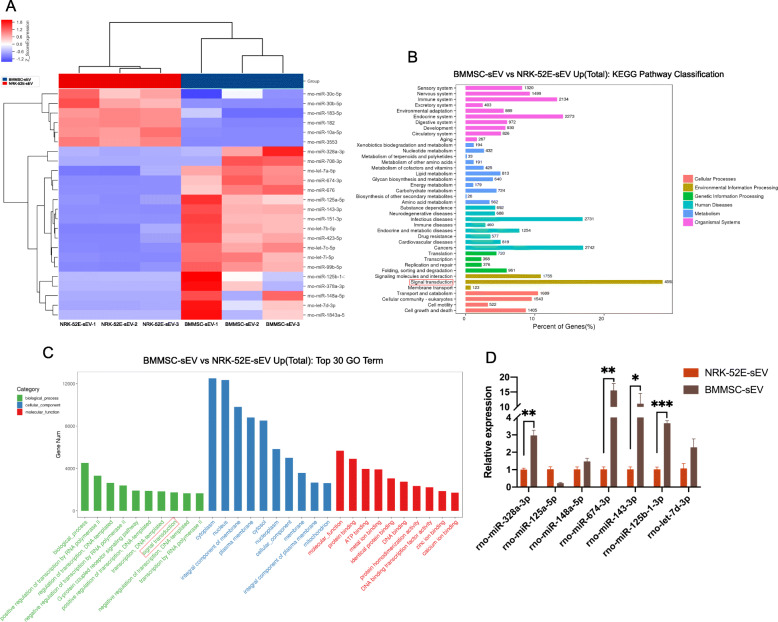
Identifying miRNAs enriched in BMMSC-sEV. **a** Heat map of differentially expressed miRNAs between BMMSC-sEV and NRK-52E-sEV showed that a total of 18 miRNAs were abundant in BMMSC-sEV. **b** KEGG pathway classification of upregulated miRNA target genes. **c** Top 30 enriched GO terms of enriched miRNA target genes. **d** qRT-PCR analysis showed that four of seven abundant miRNAs in BMMSC-sEV were in accordance with miRNA sequencing, including rno-miR-328a-3p, rno-miR-143-3p, rno-miR-125b-1-3p, and rno-miR-674-3p. *p < 0.05, **p < 0.01, ***p < 0.001, ****p < 0.0001

According to high-throughput sequencing, there were 24 differentially expressed miRNAs between BMMSC-sEV and NRK-52E-sEV. In detail, 18 miRNAs were significantly upregulated in BMMSC-sEV, while 6 miRNAs in BMMSC-sEV were downregulated compared with those in NRK-52E-sEV (Fig. 5a). Then, the expression of seven abundant miRNAs in BMMSC-sEV was detected by qRT-PCR, including rno-miR-148a-5p, rno-miR-125a-5p, rno-miR-328a-3p, rno-miR-674-3p, rno-miR-143-3p, rno-miR-125b-1-3p, and rno-let-7d-3p. The results showed that 4 of 7 were in accordance with miRNA sequencing, including rno-miR-328a-3p, rno-miR-143-3p, rno-miR-125b-1-3p, and rno-miR-674-3p (Fig. 5d). miRanda was used to predict the target genes of the abundant miRNAs in BMMSC-sEV and identified 18,903 target genes. Then, according to GO function enrichment analysis, the target genes referred to 1261 biological process entries, 2534 cellular component entries, and 1595 molecular function entries (Fig. 5c). The KEGG pathway analysis showed that 4592 predicted target genes participated in signal transduction (Fig. 5b).

### BMMSC-sEV-derived miR-328a-3p regulates the TGF-β1 pathway via Sirt7

**Fig. 6:**
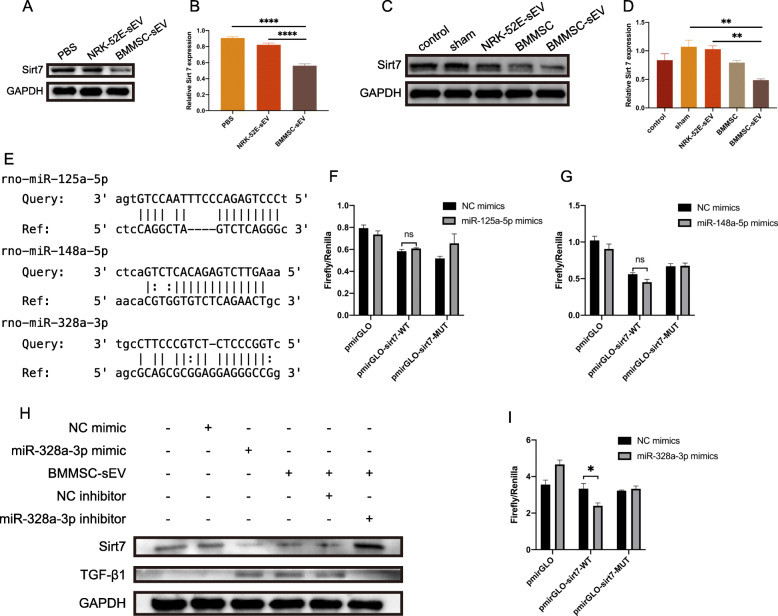
BMMSC-sEV-derived miR-328a-3p regulates the TGF-β1 pathway via Sirt7. **a** Western blot analysis showed the expression level of Sirt7 in vitro. **b** Quantified data shown in **a**. **c** The expression level of Sirt7 in vivo. **d** Quantification of **c**. **e** Schematic diagram showing the putative rno-miR-148a-5p, rno-miR-125a-5p, and rno-miR-328a-3p binding sites in Sirt7. **f**, **g**, **i** A dual-luciferase reporter assay was performed to detect the ratio of firefly luciferase activity/Renilla luciferase activity. **h** The expression levels of Sirt7 and TGF-β1 in fibroblasts incubated with PBS, the NC mimic, the miR-328a-3p mimic, BMMSC-sEV, the NC inhibitor, or the miR-328a-3p inhibitor. *p < 0.05, **p < 0.01, ***p < 0.001, ****p < 0.0001

miRNAs mediate gene silencing to negatively regulate target genes, so we detected the upstream signaling molecules of TGF-β1. The results showed that BMMSC-sEV downregulated Sirt7 in fibroblasts and in SUI rats (Fig. 6a–d), which is an upstream signaling molecule of TGF-β1 [34]. According to the target gene prediction, we found that three of the upregulated miRNAs in BMMSC-sEV were related to Sirt7 and included rno-miR-148a-5p, rno-miR-125a-5p, and rno-miR-328a-3p.

To explore the relationship between these three miRNAs and Sirt7, we performed a dual-luciferase reporter assay. The results showed that in HEK-293T cells, the luciferase activity of SIRT7-WT cotransfected with the miR-328a-3p mimic was significantly inhibited compared to that of SIRT7-WT cotransfected with the NC mimic. Moreover, the luciferase activity of SIRT7-MuT was not different between the miR-328a-3p mimic and the NC mimic. Neither the luciferase activity of the miR-148a-5p mimic nor the miR-125a-5p mimic cotransfected with SIRT7-WT changed (Fig. 6e–g, i). Collectively, these data indicated that Sirt7 was a target of miR-328a-3p. In addition, qRT-PCR results showed that in the three miRNAs, only rno-miR-328a-3p was upregulated within BMMSC-sEV. These pieces of evidence indicated that BMMSC-sEV miR-328a-3p bound to Sirt7.

To further confirm whether miR-328a-3p represses the expression of Sirt7 to mediate the TGF-β1 pathway, we cotransfected fibroblasts with the miR-328a-3p mimic or the miR-328a-3p inhibitor and BMMSC-sEV. Western blot analysis showed that the miR-328a-3p mimic repressed Sirt7 expression and enhanced TGF-β1 expression. Moreover, when fibroblasts were cotransfected with the miR-328a-3p inhibitor and BMMSC-sEV, the inhibitor and sEV had no effect on Sirt7 and TGF-β1 (Fig. 6h).

## Discussion

Stem cells have been diffusely studied and applied in various fields. Recent intriguing evidence reveals that sEV secreted by stem cells are a part of a new and essential pathway for maintaining organ homeostasis and repairing injured tissues. Some MSC-sEV have been registered in clinical trials, such as chronic diseases, immunity diseases, and acute ischemic stroke [35, 36]. sEV play a major role in cell-to-cell communication [37–39]. In addition, treatment with sEV has more advantages for tissue repair than cell therapy because it potentially avoids the safety problems associated with cell transplantation, such as immunological rejection and the possibility of malignant transformation [40]. Thus, sEV seem to represent a more effective and safer therapeutic strategy than stem cells.

Over the years, it has been extensively confirmed that BMMSC can be used for the treatment of SUI [41, 42]. Dissaranan et al. reported that the injection of BMMSC or their concentrated conditioned medium could lead to similar therapeutic effects on SUI rats [43], and Deng et al. demonstrated the equal effectiveness of BMMSC and their concentrated conditioned medium on the recovery of a dual muscle and nerve injury SUI model; the authors inferred that BMMSC secreted paracrine factors to facilitate the improvement of the urethra [43, 44]. Recent studies have verified that stem cells mainly secrete extracellular vesicles to induce changes in recipient cells and tissues [45, 46]. However, knowledge about the exact mechanism of the stem cell paracrine effects in SUI is scarce. In our present study, we further illuminated that BMMSC-derived sEV could promote ECM reconstruction to correct SUI by delivering miR-328a-3p to regulate the Sirt7/TGF-β1 signaling pathway.

The LPP test and tissue staining analysis consistently showed better recovery of urethral function and structure in the BMMSC-sEV-treated group and BMMSC-treated group than in the sham group. Overall, the effect of BMMSC-sEV was highly similar to the effect of BMMSC; even some indicators of BMMSC-sEV were slightly better than those of BMMSC. Consequently, we can conclude that BMMSC-sEV are the main effector of BMMSC in SUI, which is consistent with the theory that stem cells achieve therapeutic effects mainly through paracrine mechanisms [46]. As for the better effects of BMMSC-sEV, we speculated that this might be related to the fact that compared to the injected BMMSC, the injected BMMSC-sEV were derived from a larger number of BMMSC.

The normal function of the urethral sphincter depends greatly on the delicate balance between the elastic force of elastic fibers and the mechanical force of collagen fibers. The metabolic imbalance of the urethral ECM can cause SUI and the destruction of urodynamics [7]. Zhang et al. reported that sEV derived from MSC facilitated ECM deposition in wound healing [47]. In our present study, we demonstrated that BMMSC-sEV increased the synthesis of ECM proteins, including elastin, collagen I, and collagen III, and restored the length and thickness of elastic and collagen fibers. This finding illustrated that BMMSC-sEV play a key role in ECM remodeling.

To further ascertain how BMMSC-sEV improve urinary function and ECM remodeling, we investigated the effect of BMMSC-sEV on the TGF-β1 signaling pathway in vivo and in vitro. TGF-β1 is a crucial regulator of fibrosis and can promote the synthesis and deposition of ECM proteins [32, 33]. In the canonical signaling pathway, TGF-β1 can bind to the type I receptor, and then Smad2 and Smad3 are phosphorylated and translocate into the nucleus to regulate gene expression. Tang et al. reported that TGF-β1/Smad signaling inhibition led to a metabolic disorder of the urethral ECM and that activation of the TGF-β1/Smad signaling pathway had therapeutic effects on SUI [11]. On the other hand, MSC have been widely suggested to be mediators of the TGF-β1 signaling pathway. In chronic wounds, MSC-conditioned medium treatment could activate the TGF-β1/Smad signaling pathway and repress fibroblast differentiation [48, 49]. In our present study, we demonstrated that BMMSC-sEV significantly enhanced the expression level of TGF-β1 and the phosphorylation level of Smad2/3 in both the short and long terms, indicating that BMMSC-sEV activate the TGF-β1 signaling pathway to promote the synthesis of ECM proteins. Considering that sEV have complex components, we further investigated the specific cargo in mediating TGF-β1 signaling.

MSC sEV perform functions on target cells largely by transferring proteins, mRNAs, and miRNAs. Recently, miRNAs in sEV have attracted much attention because they can be delivered into recipient cells and play an essential role in various biological processes [20, 50–52]. Many data suggest that there exists a sorting mechanism that controls the accumulation of specific miRNAs into sEV [53–55], and these data lead us to focus on the sEV miRNAs that contribute to distinct therapeutic effects between BMMSC-sEV and NRK-52E-sEV. To identify the BMMSC-sEV miRNAs that are involved in the regulation of TGF-β1, we examined the sEV miRNA expression profile by high-throughput sequencing. Moreover, sEV miRNAs usually negatively regulate target genes, so we detected the upstream signaling molecules that negatively regulate TGF-β1. The results showed that BMMSC-sEV downregulated Sirt7 in fibroblasts and in SUI rats. Sirt7, which belongs to the mammalian sirtuin family, is a nicotinamide adenine dinucleotide oxidized form (NAD+)-dependent deacetylase. Sirt7 participates in many cellular activities, such as oncogenic transformation, aging, and cellular metabolism [56]. Studies have revealed that Sirt7 can antagonize TGF-β1 signaling and Smad3 signaling to regulate the process of fibrosis [34, 57]. In addition, target gene prediction showed that miR-148a-5p, miR-125a-5p, and miR-328a-3p, which were specifically enriched in BMMSC-sEV, were related to Sirt7. A dual-luciferase reporter assay further confirmed that Sirt7 was the target of rno-miR-328a-3p but not the target of rno-miR-148a-5p and rno-miR-125a-5p. Finally, we demonstrated that transduction of the miR-328a-3p mimic repressed the expression of Sirt7 and enhanced the expression of TGF-β1, while the miR-328a-3p inhibitor reversed the regulation of Sirt7 and TGF-β1 induced by BMMSC-sEV, which is consistent with the effect of miRNA regulation of translation repression [58]. Thus, BMMSC-sEV antagonize Sirt7 to regulate the TGF-β1 signaling pathway via miR-328a-3p.

Moreover, BMMSC-sEV could be readily internalized by fibroblasts and periurethral tissue, indicating BMMSC-sEV as a proper cargo for bioactive molecules. It is noteworthy that the fluorescence of DiI remained longer than DiI-labeled sEV in vivo. As a long-term tracer, DiI could exist in vivo for a long time. Noory et al. reported that DiI-labeled human menstrual blood stem cell-derived granulosa cells were observed in a rat model of premature ovarian failure 1 month after injection [59]. DiI only take 5–20 min to label cells; thus, it will quickly insert into the cell membrane after a periurethral injection and then diffuses slowly along the membrane. sEV are usually uptaken into the cells via the endocytic pathway and are degraded and/or recycled faster. Therefore, the persistence of DiI outlasts the labeled sEV in vivo.

One limitation of the present study was the lack of a validation experiment to verify the positive impact of the activation of TGF-β1 signaling on ECM deposition in SUI. However, considering that the TGF-β1 signaling pathway involved in the regulation of ECM metabolism has been confirmed by several previous studies, we believe that TGF-β1 is at least an important pathway of BMMSC-sEV to promote the repair of damaged urethra.

## Conclusion

In conclusion, we confirmed BMMSC-sEV as an effective treatment for SUI and demonstrated that BMMSC-sEV miR-328a-3p represses Sirt7 expression to promote ECM remodeling of damaged urethral sphincters by activating the TGF-β1 signaling pathway.

## Supplementary information

**Additional file 1 **: **Figure S1.** Characterization of fibroblasts and BMMSC. **(A)** Primary human fibroblasts derived from the vaginal wall of female SUI patients were adherent and shaped as long spindles. Scale bar, 100 μm. **(B)** HE staining of primary human fibroblasts. Scale bar, 100 μm. **(C)** Vimentin staining of primary human fibroblasts. Scale bar, 100 μm. **(D)** Under the microscope, the morphology of BMMSC was spindle-shaped and long fusiform-shaped, but few cells were polygonal-shaped. Scale bar, 100 μm. **(E)** Flow cytometry showed that 99.50% of BMMSC at passage 3 expressed CD90 on the surface, but less than 0.2% of BMMSC expressed CD34 and CD45.

**Additional file 2 **: **Figure S2**. In vivo tracking of DiI-labeled sEV. **(A)** After rats were treated with PBS, DiI, or DiI-labeled sEV, fluorescence images were taken on days 1, 2, 3, 5, 7, 10, and 14. **(B)** In DiI-labeled sEV-injected rats, the fluorescence signal intensity decreased slowly from day 1 to day 7. On day 7, 39.44% of the fluorescence was eliminated. A total of 96.14% of the fluorescence signal disappeared on day 10, and no signal was detected on day 14. The fluorescence signal intensity in the DiI group was barely attenuated from day 1 to day 14. No fluorescence signal was detected in the PBS group.

## Data Availability

The authors confirmed that all data in this study are fully available and could be obtained from the corresponding authors by reasonable requests.

## Abbreviations

SUI: Stress urinary incontinence
BMMSC: Bone marrow mesenchymal stem cells
sEV: Small extracellular vesicles
ECM: Extracellular matrix
TGF-β1: Transforming growth factor β1
p-Smad2/3: Phosphorylation of Smad2/3
HE: Hematoxylin and eosin
EVG: Elastic van Gieson
α-SMA: Alpha-smooth muscle actin
GO: Gene Ontology
KEGG: Kyoto Encyclopedia of Genes and Genomes
FBS: Fetal bovine serum
PBS: Phosphate-buffered saline
GAPDH: Glyceraldehyde-3-phosphate dehydrogenase
LPP: Leak point pressure
DAPI: 4′,6-Diamidino-2-phenylindole
DiI: 1,1′-Dioctadecyl-3,3,3′,3′-tetramethylindocarbocyanine perchlorate
NRK-52E: Normal rat kidney 52E cells
HEK-293T: Human embryonic kidney 293T cells
NTA: Nanoparticle tracking analysis
