# Supplementary figures and images for: BMMSC-sEV-derived miR-328a-3p promotes ECM remodeling of damaged urethral sphincters via the Sirt7/TGFβ signaling pathway
Source: Stem Cell Res Ther. 2020 Jul 16;11:286. doi: 10.1186/s13287-020-01808-2 (PMC7364490; doi:10.1186/s13287-020-01808-2)

**A**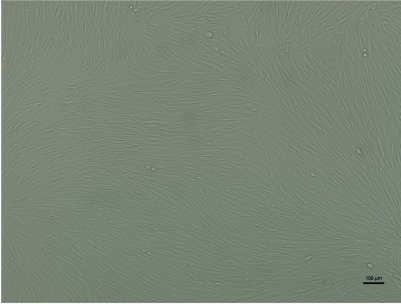**B**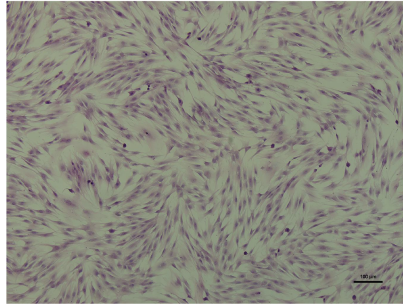**C**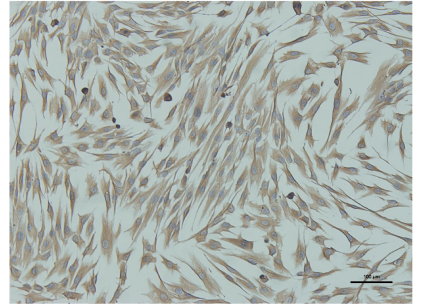**D**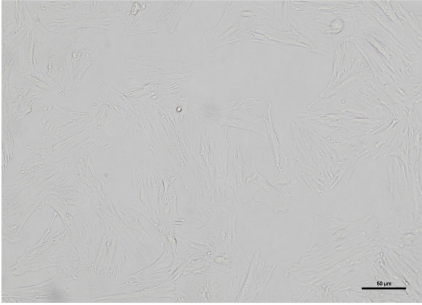**E**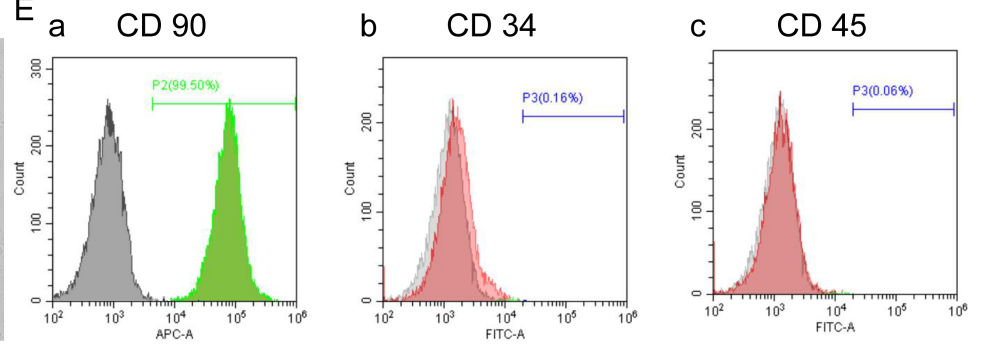

Supplement: Supplementary file 1 — Additional file 1 : Figure S1. Characterization of fibroblasts and BMMSC. (A) Primary human fibroblasts derived from the vaginal wall of female SUI patients were adherent and shaped as long spindles. Scale bar, 100 μm. (B) HE staining of primary human fibroblasts. Scale bar, 100 μm. (C) Vimentin staining of primary human fibroblasts. Scale bar, 100 μm. (D) Under the microscope, the morphology of BMMSC was spindle-shaped and long fusiform-shaped, but few cells were polygonal-shaped. Scale bar, 100 μm. (E) Flow cytometry showed that 99.50% of BMMSC at passage 3 expressed CD90 on the surface, but less than 0.2% of BMMSC expressed CD34 and CD45. [file 13287_2020_1808_MOESM1_ESM.pdf]

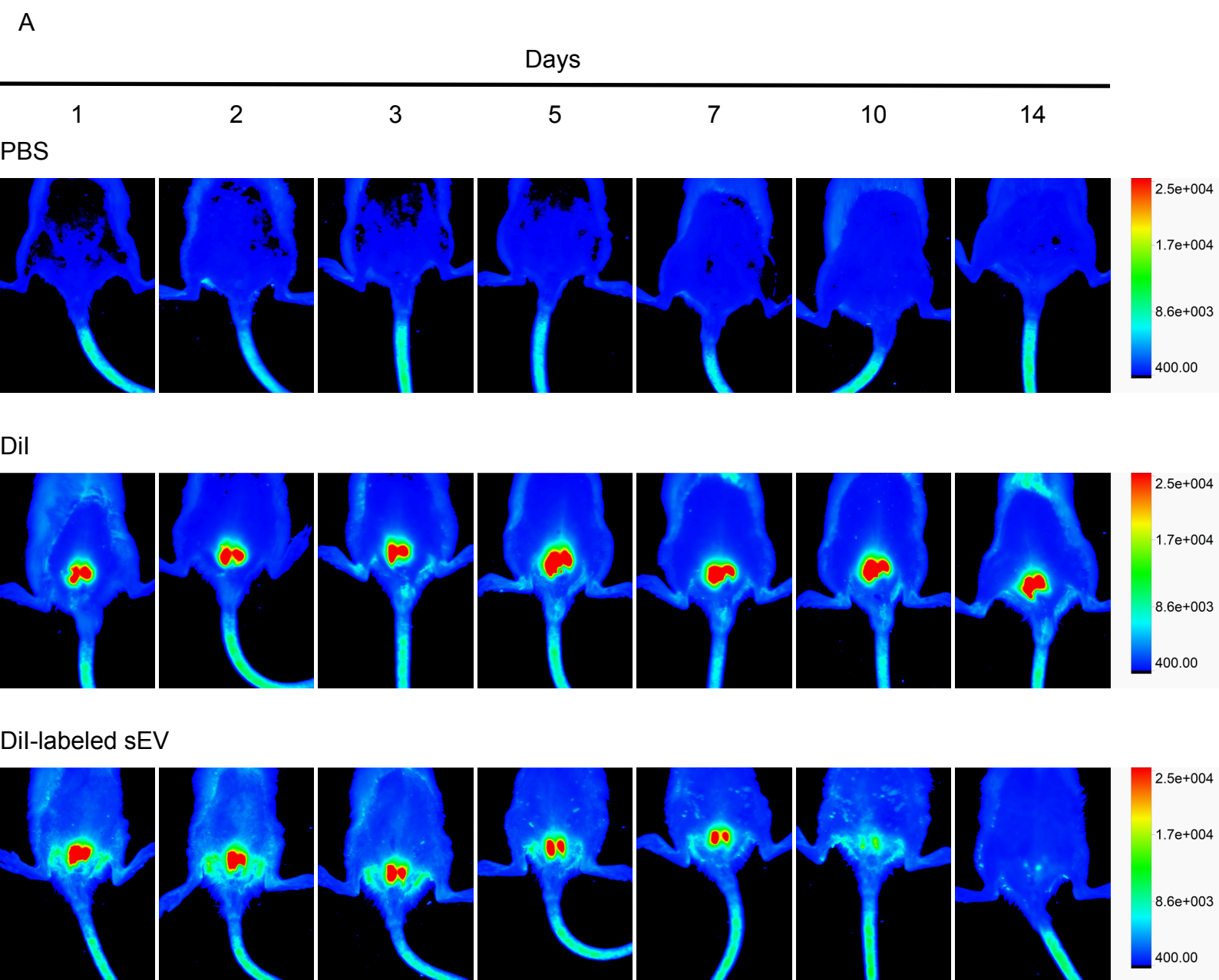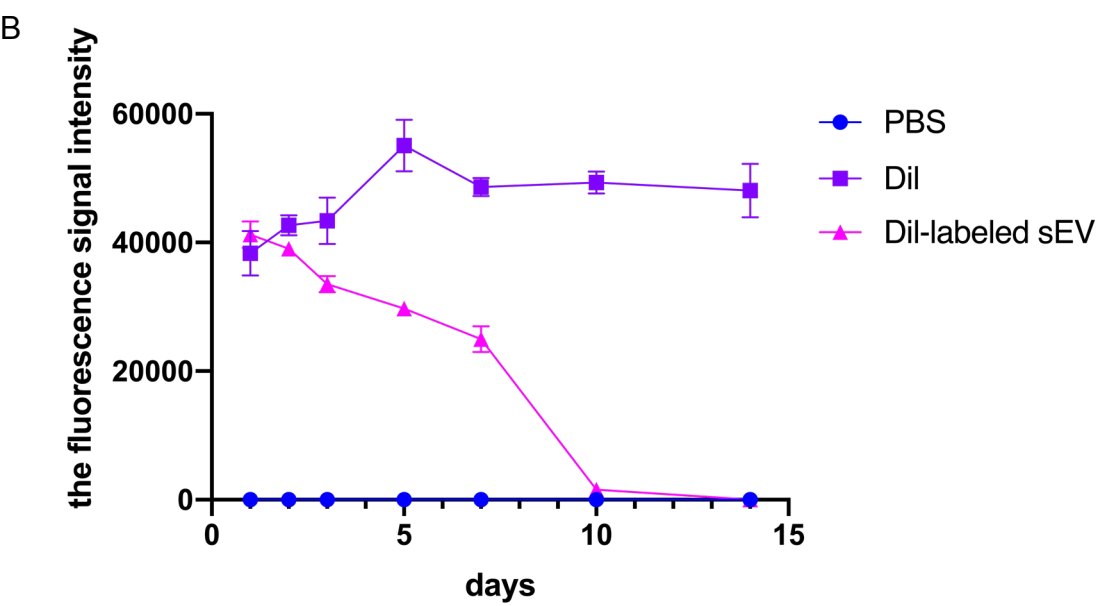

Supplement: Supplementary file 2 — Additional file 2 : Figure S2. In vivo tracking of DiI-labeled sEV. (A) After rats were treated with PBS, DiI, or DiI-labeled sEV, fluorescence images were taken on days 1, 2, 3, 5, 7, 10, and 14. (B) In DiI-labeled sEV-injected rats, the fluorescence signal intensity decreased slowly from day 1 to day 7. On day 7, 39.44% of the fluorescence was eliminated. A total of 96.14% of the fluorescence signal disappeared on day 10, and no signal was detected on day 14. The fluorescence signal intensity in the DiI group was barely attenuated from day 1 to day 14. No fluorescence signal was detected in the PBS group. [file 13287_2020_1808_MOESM2_ESM.pdf]
